# Acute Liver Failure Due to *Plasmodium falciparum*: A Rare Case from a Non-Endemic Country

**DOI:** 10.5152/tjg.2026.25669

**Published:** 2026-03-23

**Authors:** Ersin Batıbay, Osman Yüksekyayla, Mahmut Polat

**Affiliations:** Department of Gastroenterology, Harran University Faculty of Medicine, Şanlıurfa, Türkiye

To the Editor,

A 34-year-old male presented with jaundice, fever, and altered mental status. He had recently returned from Tanzania without chemoprophylaxis. He also reported experiencing night sweats after his return, raising suspicion for an imported infectious disease.

At admission, AST was 130 U/L, ALT 148 U/L, and total bilirubin 18.5 mg/dL (direct 12.8 mg/dL). INR was 1.51, and platelet count was 28 × 10^9^/L. LDH was elevated with low haptoglobin, consistent with intravascular hemolysis. White blood cell count was 5.5 × 10^9^/L with neutrophil predominance (74%) and lymphopenia (7%). Hemoglobin was 14.6 g/dL. Viral hepatitis markers (HBsAg, anti-HBe, anti-HCV, and anti-HAV IgM) and HIV were negative. HBV DNA and HCV RNA were undetectable. Autoimmune hepatitis markers were also negative. Additional infectious workup, including Widal, Brucella testing, VDRL, and TORCH, was unremarkable. The patient reported no alcohol consumption, no use of prescription or over-the-counter medications, and no exposure to toxic or environmental hepatotoxic agents.

Abdominal ultrasound showed normal a liver size and echotexture and with no biliary dilation. The intrahepatic and extrahepatic ducts were normal. Chest radiograph demonstrated bilateral interstitial opacities compatible with acute lung injury/acute respiratory distress syndrome (ARDS).

Acute liver failure (ALF) was present at admission. Acute kidney injury developed on day 2. By day 3, he developed grade 2 hepatic encephalopathy and ARDS. Hemodialysis was required until day 10. Peripheral smears revealed abundant *Plasmodium falciparum* trophozoites (Figure 1). PCR confirmed the diagnosis, and results were independently verified by the regional public health laboratory. The patient received intravenous artesunate followed by oral artemether–lumefantrine. Five sessions of plasmaphresis sessions were performed on days 2, 3, 5, 6, and 7. Parasitemia cleared by day 4, as confirmed by negative peripheral blood smears (Figure 2). Encephalopathy resolved by day 14. Liver tests improved steadily and were near normal by week 3. The patient was discharged on day 30. Full biochemical recovery was documented by week 8. Written informed consent was obtained.

Hepatic dysfunction is frequent in malaria, but progression to ALF is uncommon. Several mechanisms contribute to liver injury including: microvascular sequestration of parasitized erythrocytes, endothelial dysfunction, reduced hepatic oxygen delivery, hemolysis, cholestasis, and oxidative stress.^1^^,^^2^ These overlapping mechanisms may result in mixed hepatocellular and cholestatic biochemical profiles. Recognizing such patterns is essential when evaluating ALF in returning travelers.

Recent hepatology literature highlights the clinical value of interpreting biochemical injury patterns, as variable hepatocellular and cholestatic profiles may help guide diagnostic reasoning in acute liver failure. This broader perspective is useful for identifying unusual causes of ALF, including severe malaria.

Severe systemic insults frequently involve multiple organs. Nationwide mushroom intoxication data from Türkiye have shown that acute hepatotoxic injuries often coexist with renal and respiratory dysfunction.^3^ Although the etiologies differ, this multisystem pattern resembles the clinical course observed in severe *P. falciparum* infection.

Clinical outcomes in *P. falciparum*–associated ALF vary. Some reports demonstrate full recovery when treatment is initiated promptly, whereas others report high mortality despite therapy.^4^^-^^6^ Parasitemia burden, timing of artesunate administration, and access to supportive therapies influence prognosis.

In the present case, plasmapheresis was used as an adjunctive supportive therapy in the setting of severe multiorgan dysfunction, including acute liver failure, acute kidney injury, and ARDS. Although plasmapheresis is not a standard treatment for malaria, it has been reported as a rescue strategy in selected cases of severe malaria and systemic inflammatory states, with the aim of reducing circulating inflammatory mediators, hemolysis-related products, and parasite-derived toxins.^7^

In patients presenting with ALF after recent travel to endemic regions, *P. falciparum* infection should remain a key consideration. Early parasitologic confirmation and immediate antimalarial therapy are crucial for survival.

## Figures and Tables

**Figure 1.**
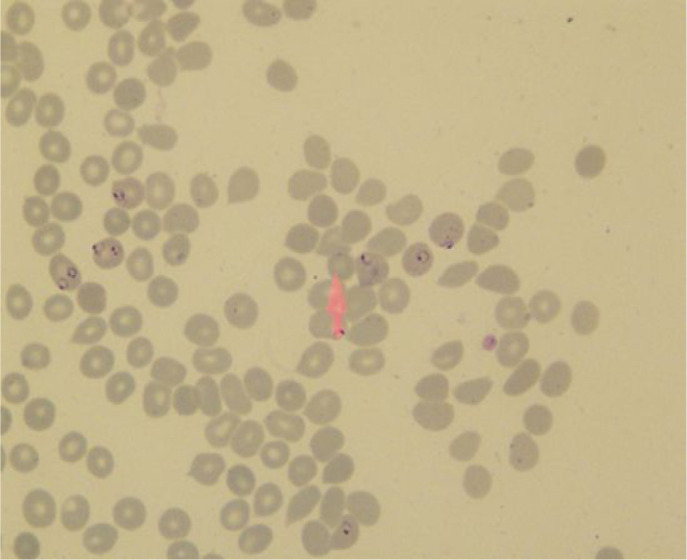
Peripheral blood smear showing multiple ring forms of *Plasmodium falciparum.*

**Figure 2.**
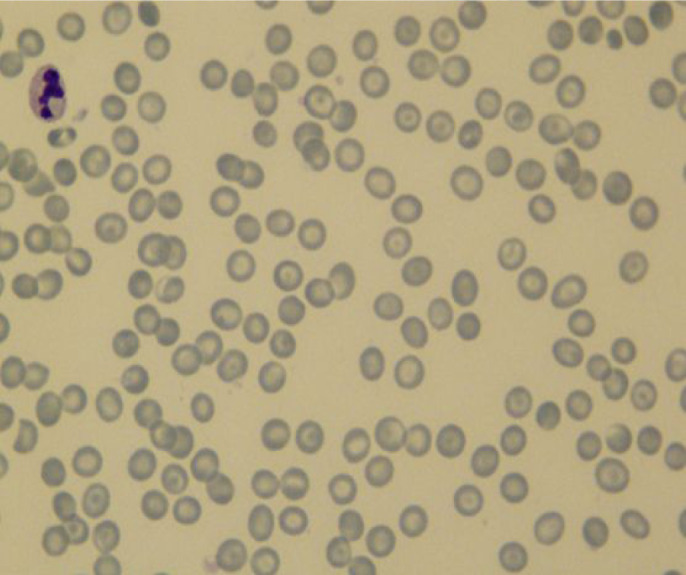
Negative peripheral smear after antimalarial treatment (Day 5).

## Data Availability

The data that support the findings of this study are available on request from the corresponding author.
